# The Intersection of Purine and Mitochondrial Metabolism in Cancer

**DOI:** 10.3390/cells10102603

**Published:** 2021-09-30

**Authors:** Humberto De Vitto, Danushka B. Arachchige, Brian C. Richardson, Jarrod B. French

**Affiliations:** Division of Nucleotide Metabolism & Drug Discovery, The Hormel Institute, University of Minnesota, 801 16th Avenue NE, Austin, MN 55912, USA; hdevitto@umn.edu (H.D.V.); arach021@umn.edu (D.B.A.); rich1952@umn.edu (B.C.R.)

**Keywords:** purines, mitochondrial metabolism, amino acids, metabolic reprogramming, cancers

## Abstract

Nucleotides are essential to cell growth and survival, providing cells with building blocks for DNA and RNA, energy carriers, and cofactors. Mitochondria have a critical role in the production of intracellular ATP and participate in the generation of intermediates necessary for biosynthesis of macromolecules such as purines and pyrimidines. In this review, we highlight the role of purine and mitochondrial metabolism in cancer and how their intersection influences cancer progression, especially in ovarian cancer. Additionally, we address the importance of metabolic rewiring in cancer and how the evolving landscape of purine synthesis and mitochondria inhibitors can be potentially exploited for cancer treatment.

## 1. Purine Metabolism

**Figure 1:**
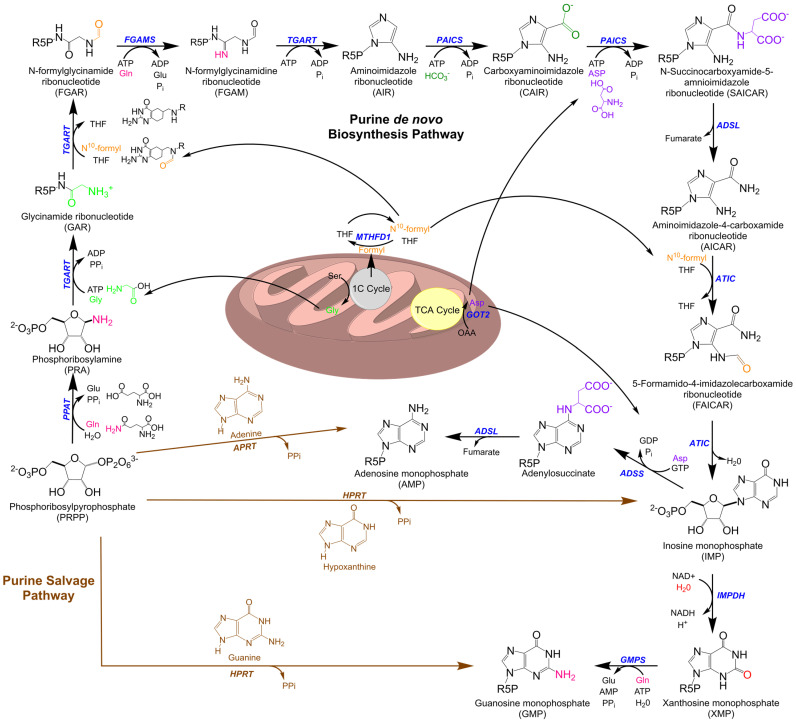
Purine metabolic pathways. The schematic representation shows the de novo and salvage pathways and their crosstalk with mitochondria. The conserved de novo biosynthesis pathway to generate IMP consists of 10 chemical steps catalyzed by 6 gene products in humans. These include the trifunctional enzyme *TGART*, composed of GAR synthetase (GARS), GAR transformylase (GARTfase), and AIR synthetase (AIRS) domains; the bifunctional enzymes *PAICS*, composed of CAIR synthetase/AIR carboxylase (CAIRS) and SAICAR synthetase (SAICARS), and *ATIC*, composed of AICAR transformylase (AICART) and IMP cyclohydrolase (*IMPCH*); and three monofunctional enzymes, phosphoribosyl amidotransferase (*PPAT*), formylglycinamidine ribonucleotide synthetase (*FGAMS*), and adenylosuccinate lyase (*ADSL*). Downstream IMP is converted to (1) GMP through stepwise reactions of IMP dehydrogenase (*IMPDH*) followed by GMP synthetase (*GMPS*) and (2) AMP via adenylosuccinate synthetase (*ADSS*) followed by *ADSL*. The salvage pathway requires PRPP to generate IMP and GMP through one-step reactions mediated by hypoxanthine phosphoribosyltransferase (*HPRT*) utilizing hypoxanthine and guanine bases. AMP is generated by adenine phosphoribosyltransferase (*APRT*) utilizing adenine base and PRPP as substrates. Mitochondria supply precursors for purine de novo biosynthesis including glycine, N^10^-formyl THF, and aspartic acid through their one-carbon cycle (1C cycle) and tricarboxylic acid cycle (TCA).

Nucleotides, beyond forming the fundamental building blocks of the genetic code and its expression, serve a variety of active biochemical roles in the biology of the cell. Consisting of either a single-ring pyrimidine or a fused-ring purine carbon–nitrogen nucleobase and a ribose/deoxyribose-phosphate moiety, nucleotides and their derivatives act as energy carriers to drive enzymatic reactions, mediate signaling within and between cells, and play numerous vital roles in the regulation of metabolism beyond their own homeostasis [1,2]. Due to the central nature of nucleotides in cellular function, it is critical that cells maintain an uninterrupted supply of both pyrimidines and purines. In mammals, this is achieved for purines by two main pathways, salvage from existing bases and de novo biosynthesis, with significant activity occurring in the liver [3]. Under normal physiological conditions, most of the purine pool is generated by the salvage pathway with the nucleic acid breakdown process, leading to the release of free purine nucleobases in the form of adenine, guanine, and the hypoxanthine base of inosine monophosphate (IMP) [4]. These free bases are attached to phosphoribosyl pyrophosphate (PRPP) to form purine nucleoside monophosphates by either adenine phosphoribosyltransferase (*APRT*), which mediates adenosine monophosphate (AMP) formation, or hypoxanthine-guanine phosphoribosyltransferase (*HGRT*), which acts on hypoxanthine to form IMP and guanine to form guanosine monophosphate (GMP) (Figure 1) [5]. As a critical precursor of de novo biosynthesis, PRPP plays an important role in maintaining both de novo biosynthetic and salvage pathways, with the distribution and balance of the nucleotide pool in each cell type being vital for regular cellular activities [6].

Under high cellular purine demands exceeding the capacity of salvage, the nucleotide requirement is met by upregulation of the de novo biosynthetic pathway [4]. This is a highly conserved pathway that produces AMP and GMP using metabolic precursors including PRPP, several amino acids (glutamine, aspartic acid, and glycine), small molecule cofactors N^10^-formyl Tertrahydrofolate (THF) and Nicotinamide Adenine Dinucleotide (NAD^+^), and existing adenosine triphosphate (ATP) and guanosine triphosphate (GTP). In humans, de novo biosynthesis requires a sequence of ten distinct reactions catalyzed by six enzymes. Three of these are multifunctional enzymes catalyzing multiple steps in the pathway, comprising the two bifunctional enzymes phosphoribosylaminoimidazole carboxylase (*PAICS)* and AICAR transformylase/inosine monophosphate cyclohydrolase (*ATIC)* and the trifunctional enzyme glycinamide ribonucleotide transformylase (*TGART)* [7,8]. When active, the pathway is limited both by substrate availability and by the reaction rate of its initial step, the conversion of PRPP to phosphoribosylamine (PRA) by phosphoribosylpyrophosphate amidotransferase (*PPAT*) [9]. The final product of the de novo biosynthesis pathway, IMP, is the precursor for both AMP and GMP, which are formed via two further enzymatic reactions; in total, the energy from hydrolysis of six ATP molecules to adenosine diphosphate (ADP) is required to synthesize one molecule of IMP from PRPP, whereas nucleotide salvage is not dependent on stored phosphate bond energy.

As the presumed primary rate-limiting step of purine biosynthesis, *PPAT* activity is tightly regulated. *PPAT* possesses two nucleotide-binding sites near the active site, allowing for feedback control by downstream purine nucleotides via allosteric inhibition [10,11]. Furthermore, there is a growing body of evidence that signaling pathway enzymes such as protein kinase B (*PKB*) and ribosomal protein S6 kinase (*S6K*) influence IMP production directly through the phosphorylation of purine biosynthetic enzymes [12]. One such modification is the Thr397 phosphorylation of *PPAT* by PKB, detected in purine supplemented conditions only and affecting downstream inosine monophosphate (IMP) production [13]. Similarly, as the limiting metabolic input, regulation of PRPP levels affects the rate of purine synthesis. Analysis of PRPP in different growth stages in HTC116 colon cancer cells demonstrated that rates of purine synthesis via both salvage and the de novo pathways increased by 5 and 3.3 fold, respectively, from the end of the G1 phase to the beginning of the S phase, with the de novo increase attributed to an increase in intracellular phosphate stimulating PRPP synthetase activity [14]. More broadly, high-throughput global proteomic studies have revealed 174 post-translational modifications within the six enzymes across the purine de novo biosynthetic pathway [13]. 

In an additional mechanism of pathway regulation, still incompletely understood, purine de novo synthesis enzymes cluster into large complexes termed purinosomes, presumably to increase pathway efficiency and isolate reactive intermediates. A purinosome core of *PPAT*, *TGART*, and formylglycinamidine ribonucleotide synthetase (*FGAMS*) interacts with *PAICS*, adenylosuccinate lyase (*ADSL*), and *ATIC*, which also transiently interact with each other, suggesting the possibility of association-dependent regulation of the pathway as a whole [15]. Furthermore, super-resolution microscopy studies demonstrate that these purinosomes colocalize with mitochondria, potentially positioning them in areas of high ATP and metabolite concentrations to promote forward flux through the pathway [16]. 

## 2. Purine Metabolism in Cancer

The defining distinction between neoplastic cells and their normal counterparts is the unregulated and increased rate of growth of the former [17]. This requires their metabolism to be altered by oncogenes and loss of tumor suppressors to support the synthesis of available nutrients into cellular biomass [18]. The first historical evidence that cancer cells adapt their metabolism to support cell growth was the so-called “Warburg effect”, reporting increased aerobic glycolysis in cancer cells with a corresponding increase in glucose uptake and lactate excretion [19,20]. Similar behaviors are observed in other anabolic pathways, with upregulation of nucleotide, protein, and lipid uptake and biosynthesis associated with increased cell growth [17]. In particular, nucleotide synthesis is a frequently limiting factor of proliferation: de novo nucleotide biosynthesis is an energy-intensive process, is dependent on sources of carbon and nitrogen, and requires multiple inputs distributed between multiple pathways and organelles, especially mitochondria [21,22]. As such, nucleotide biosynthesis and its associated mitochondrial pathways have been targeted by chemotherapeutic regimens for decades. The typical approach is by direct inhibition of this pathway using purine antimetabolites, analogs of nucleotides, or their precursors acting as competitive inhibitors. These have proven to be effective treatments acting to stall DNA replication or cause apoptosis via DNA damage, and they reflect a significant percentage of currently available cancer treatments [23]. Historically, 6-mercaptopurine and 6-thioguanine, both purine analogs, were the first drugs to be used clinically for the treatment of leukemias [24]. Another class of purine antimetabolites, the purine deoxynucleoside analogs fludarabine, cladribine, clofarabine, nelarabine, and pentostatin, are US Food and Drug Administration (FDA)-approved agents for the treatment of cancers (Table 1) [23]. Notably, however, none of these currently approved treatments target purine biosynthesis directly; instead, they target upstream input availability and downstream utilization of synthesized purines. While several biosynthetic inhibitors are in development, a better understanding of the precise molecular mechanisms of these agents and identification of new enzyme and metabolite targets is crucial for improving options for treatment [25].

### Purine Metabolic Enzymes and Intermediates

In accordance with the expected increased rate of nucleobase synthesis, elevated concentrations of purine metabolites are common to most if not all forms of cancer, an observation leading to the use of purine antimetabolites as early treatments to inhibit cell growth. Similarly, the activity of the de novo purine biosynthetic enzymes are typically dysregulated in cancers, including *PPAT* in lung cancer; *GART*, *ATIC*, and *GMP synthase* (*GMPS*) in liver cancer; *PAICS* in bladder, prostate, colon, lung cancer, and neuroblastoma; *ADSL* in endometrial cancer and in triple-negative breast cancer; and *inosine-5′-monophosphate dehydrogenase* (*IMPDH*) in glioblastoma and liver and lung cancer [33,34,35,36,37,38,39,40,41,42,43]. At the clinical level, analyses studying large-scale patient cohorts and different cancer types identify the regulation of purine biosynthetic enzymes as a predictor of clinical outcome. Notably, a meta-analysis of multiple cancer cohorts identified elevated expression levels of *PPAT* as a strong predictor of tumor malignancy, altering nitrogen metabolism to favor purine biosynthesis for cell proliferation [33]. A similar cohort study of hepatocellular carcinoma (HCC) identified dysregulation of *IMPDH* expression as a key contributor to cancer progression and predictor of clinical outcome [43]. 

As potential additional targets for treatment, the effects of elevated purine metabolites are not limited to increasing the rate of DNA and RNA synthesis. While the complex roles played in regulation by purine nucleotide triphosphates are too numerous to detail here, two key examples have particular relevance to oncogenesis. Signaling by small G-proteins is sensitive to intracellular GTP/GDP levels, the reduction of which inhibits the mechanistic target of rapamycin C1 (*mTORC1*) activity via its regulator Rheb and hence decreases tumor growth in mice bearing non-small-cell lung cancer (NSCLC) xenografts model [44]. Similarly, the production of GMP is crucial for generating cyclic guanosine monophosphate (cGMP) to promote cGMP-dependent protein kinases to activate the mitogen-activated protein kinase (*MAPK*) pathway, resulting in the characteristic increase in stem cell gene expression that leads to breast cancer lung metastasis [45].

With respect to metabolic intermediates, N-succinocarboxyamide-5-aminoimidazole ribonucleotide (SAICAR) is of particular interest as an allosteric regulator of pyruvate kinase, isoform 2 (*PKM2*), a glycolytic enzyme also commonly upregulated in oncogenesis [4,46]. Together with serine, SAICAR induces protein kinase activity of *PKM2* for sustained proliferative signaling of cancer cells as a response to their inherent glucose limitation [47,48]. Notably, SAICAR accumulation is not observed in normal epithelial cells and fibroblasts [49]. The immediately downstream metabolite, AICAR, forms the AMP analog 5-Amino-1-βD-ribofuranosylimidazole-4-carboxamide monophosphate (ZMP) when phosphorylated. This has multiple downstream effects, including, interestingly, activation of AMP-activated protein kinase (*AMPK*). *AMPK* is a sensor of ATP levels, acting pleiotropically to increase energy storage and reduce energy usage when the ATP:AMP ratio is excessively low, though its role in cancer progression is complicated by its action in both inhibitions of growth and protection against stressors [50].

## 3. Intersections of Purine Metabolism with Broader Cancer Pathways

**Figure 2:**
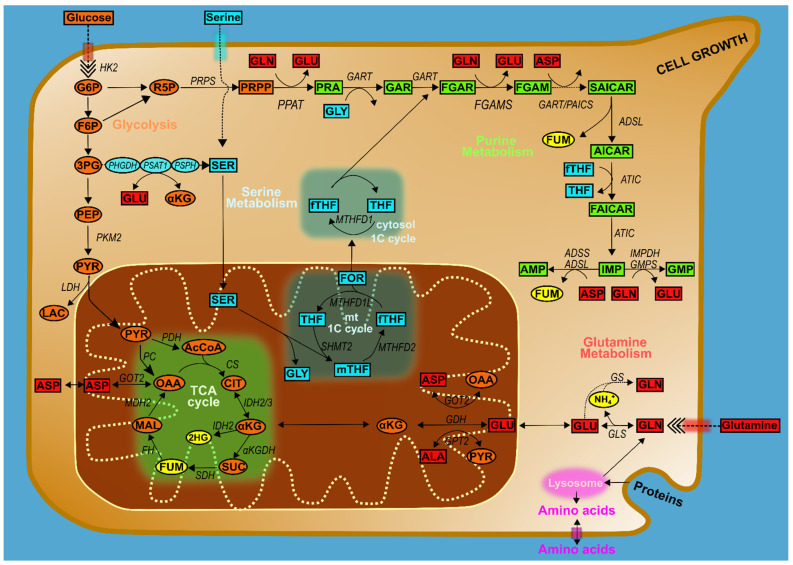
Interconnectivity of purine metabolism. The schematic representation illustrates how proliferative cells use nutrient availability and metabolic networks that feed into, are regulated by or otherwise integrated with purine metabolism. Key reactions in central metabolism are shown, including how glucose, glutamine, serine/glycine, one-carbon, and mitochondrial metabolism are involved in the de novo purine biosynthesis.

Nucleotide synthesis is only one of many biosynthetic pathways on which a cell depends for growth. Incoming substrates must be directed toward protein, lipid, and small molecule synthesis as well and are consumed for energy production by one of several pathways, and the cell possesses sophisticated biochemical circuitry to up- and down-regulate all of these various pathways according to its current needs. As cancer cells depend on altering this regulation to support abnormally rapid growth, restoring or replicating inhibitory pathways is an alternative to the direct competitive inhibition of intermediate substrates described previously [51]. With respect to purine biosynthesis, crosstalk in the form of substrate generation and enzyme regulation exists to balance this pathway with glycolysis, amino acid metabolism and associated single-carbon intermediates, and mitochondrial metabolism, all of which represent potential therapeutic targets (Figure 2) [21,52].

## 4. Glucose Metabolism and Purines

As previously noted, the increased uptake and metabolism of glucose underlying the Warburg effect is a hallmark of oncogenesis [53]. As the primary carbon source for biosynthesis, glucose metabolism is inherently linked to synthetic and metabolic pathways by directly or indirectly supplying them with substrates, and purine biosynthesis depends on the glycolytic intermediates glucose-6-phosphate (G6P), 3-phosphoglycerate (3PG), and fructose-6-phosphate (F6P), as well as the glycolytic enzyme *PKM2*. The glycolytic intermediate G6P can be rerouted into the oxidative branch of the pentose phosphate pathway (PPP), and the intermediates 3PG and F6P can be rerouted into the non-oxidative branch of the PPP to generate the sugar component of nucleic acids, ribose 5-phosphate (R5P); the latter pathway in particular is frequently upregulated in cancer lines [54]. R5P additionally can go on to be converted to PRPP, as a donor of the ribose group required for nucleotide biosynthesis. Regulation of glycolysis has been demonstrated to affect nucleotide synthesis in vivo, with the glycolytic kinase enzyme *PKM2*, a rate-limiting enzyme in glycolysis, supporting the process and the resulting cell proliferation in primary mouse embryonic fibroblasts [55]. Under allosteric regulation by endogenous and exogenous activators and inhibitors including the nucleotide intermediate SAICAR, inhibition of *PKM2* markedly decreases the glycolytic rate, allowing the generation of glycolytic intermediates that fuel into purine biosynthesis [56]. While the pleiotropic effects of PKM2 make it difficult to dissect its mechanisms of action in a given cancer line, thus making its suitability for cancer treatment similarly difficult to determine [57,58], it and its associated pathways represent an excellent target for further research.

### 4.1. Amino Acids, One-Carbon Metabolism, and Purines

Many of the interactions of cellular biosynthetic pathways are mediated by amino acid intermediates acting as biochemical substrates in lieu of their incorporation into proteins. The de novo purine biosynthetic pathway requires several amino acids as substrates of the enzymatic process, including aspartate, serine, glycine, and glutamine, all subject to multiple pathways that therefore require strict regulation of their competing demands. Serine and glycine metabolism are closely linked and together provide essential precursors for the synthesis of macromolecules: serine is the major donor of one-carbon units, and glycine is a major source of methyl groups for the one-carbon pools required for nucleotide synthesis, methylation, and reductive metabolism [59]. Each can be imported from the extracellular space, scavenged from protein hydrolysis, or synthesized directly and can be interconverted to meet cellular demand (Figure 2). De novo biosynthesis is primarily driven by the glycolytic intermediate 3PG, which is converted to serine in a three-step enzymatic reaction. This is followed by conversion to glycine by serine hydroxymethyltransferase isoforms 1 and 2 (*SHMT1/2)* in the cytosol and mitochondria, respectively. Notably, the reactions of one-carbon metabolism occur in different cellular compartments. The mitochondrial one-carbon cycle produces glycine from serine (via *SMHT2*) to release the metabolite formate. This is exported to the cytoplasm using tetrahydrofolate (THF) as a carrier to then participate in the cytosolic one-carbon cycle and enter the de novo purine biosynthetic pathway [60,61]. This 10-fTHF cofactor transfers one carbon to purines and regulates the activity of *GART* and *ATIC*. Furthermore, glycine serves as a substrate for the conversion of PRA to glycinamide ribonucleotide (GAR) by the activity of phosphoribosylglycinamide synthetase (*GARS*) [62,63]. In the end, the synthesis of one purine requires the input of two one-carbon units and one further molecule of glycine to produce IMP. 

In rapidly proliferating cancer cells, much of the intracellular serine is converted to glycine to drive one-carbon metabolism and formate production [64], with the incorporation of a one-carbon unit from serine into nucleotides observed in cancer growth [65]. More recent studies have shown that numerous cancer cells require *SHMT2* activity for optimal proliferation and tumorigenicity [66] and that serine depletion inhibits cancer cell proliferation and decreases purine levels [67]. In line with this, in vivo studies in a mouse xenograft model indicate a therapeutic benefit of dietary depletion of serine [64]. Targeting one-carbon metabolism using anti-folates to deplete the THF cofactor is therefore a common and effective anticancer therapy (Table 1). Folate analogs including aminopterin, methotrexate, pemetrexed, and pralatexate are used for the treatment of cancers to inhibit cytosolic one-carbon metabolism enzymes including dihydrofolate reductase (*DHFR*) and thymidylate synthase (*TYMS*), with an indirect effect on de novo purine biosynthetic metabolism [27]. It must be noted, however, that folate inhibition on its own is not necessarily a panacea, given the variance seen in cancer response to dietary folate supplementation [68,69], underscoring the need for a variety of available cancer treatments.

Like serine and glycine, glutamine and aspartate can be transported into the cells via the activity of high-affinity transporters (*SLC1A5*, *GLAST*, and related proteins) or synthesized de novo. Glutamine, beyond its role in proteins, is a major carrier of nitrogen in the body for multiple cellular processes, feeding into the tricarboxylic acid cycle (TCA cycle) and directly providing nitrogen at several points in the purine biosynthetic pathway [70]. Furthermore, glutaminolysis begins with the hydrolytic deamination of glutamine to glutamate and inorganic ammonia via the activity of glutaminases (*GLSs*); this glutamate can serve as a metabolic substrate to produce aspartate via the activity of aspartate aminotransferase (*GOT1/2*) and phosphoserine via the activity of phosphoserine aminotransferase 1 (*PSAT1*), with both aspartate and serine-derived glycine being essential precursors for the de novo purine biosynthetic pathway (Figure 2). Aspartate itself can be produced by transamination of oxaloacetic acid (OAA), either in the mitochondria via *GOT2* activity or in the cytoplasm via *GOT1* activity, as part of its participation in the malate redox shuttle.

Dysregulation of glutamine enzymes and reprogramming of glutamine metabolism are characteristic of rapidly dividing cancer cells. Cancer cells under glutamine starvation undergo cell cycle arrest, which can be rescued by exogenous nucleotides [71]. Alternatively, cells can mitigate glutamine starvation via upregulating glutamine synthetase *(GS)* to reverse *GLS*-mediated deamination and recover glutamine from glutamate (and hence the TCA cycle) to promote nucleotide biosynthesis and support anabolic cell growth [72]. This metabolic reprogramming in cancer towards glutamine metabolism-regulating tumorigenesis invasion and bioenergetics has also been reported in ovarian cancer (OvCa), where highly invasive ovarian cancer cells are markedly glutamine-addicted [73]. Supporting this, under hypoxia or mitochondrial electron transport chain (ETC) dysfunction, cancer cells selectively prioritize the use of aspartate for the synthesis of nucleotides over asparagine and arginine production, draining the components of the TCA pathway to drive glutamine synthesis and hence nucleotide production [74,75]. Thus, de novo glutamine synthesis, and GS in particular, is a primary link between glutamine and purine metabolism in cancer and a potential therapeutic target.

### 4.2. The Master Regulators of Metabolism

To balance these various cellular needs during growth, as well as growth itself, the cell requires the action of centrally coordinating proteins and pathways. One of the most prominent is the Myc proto-oncogene protein (*c-Myc*) transcription factor, which directly regulates the expression and activity of up to 15% of all genes in humans, including multiple enzymes associated with de novo purine biosynthesis, glutamine metabolism, serine-derived glycine pathway, and one-carbon metabolism [76,77,78,79]. With respect to de novo purine biosynthesis, *c-Myc* binds to the bi-directional promoters of *PPAT* and *PAICS* to increase pathway throughput and support cell proliferation [77]. As a proto-oncogene and major regulator of cell proliferation, *c-Myc* in cancer reprograms glutamine metabolism to maintain pools of available nitrogen carriers, an abundant supply of aspartate, and usable energy exceeding the requirement for nucleotide biosynthesis promoting tumorigenesis [52,80]. Beyond *c-Myc*, mTOR as a central sensor of cellular reserves is also involved in purine biosynthesis, among its many functions. The mTORC1-mediated activation of transcription factor 4 (*ATF4*) regulates the expression of *MTHFD2*, which provides one-carbon units for de novo purine biosynthesis; depleting these intermediate proteins reduces incorporation of isotope-labeled glutamine nitrogen into purines [81]. Moreover, mTOR has been shown to be involved in purinosome assembly, leading to the regulation of purine metabolism by spatiotemporal control over protein association [16]. Similarly, the AMP:ATP energy sensor *AMPK*, regulated by multiple factors including the purine intermediate AICAR, in turn regulates a variety of energy-consuming cellular processes including the PPP [82,83]. More recently, the metabolic intermediate formate has been shown to indirectly repress *AMPK* by inducing a metabolic reprogramming from low to high adenine nucleotide levels, leading to an increase in glycolytic rate [84].

## 5. Purine Metabolism at the Mitochondria

With the heavy involvement of the TCA cycle and associated metabolites in purine biosynthesis, as well as its energetic needs, it is perhaps unsurprising that mitochondria as a whole are critical to the pathway. Mitochondria are the central metabolic organelle, coordinating cellular energy production and the availability of building blocks required for cell proliferation [85]. For de novo biosynthesis of purines, glutamine and aspartate are required as nitrogen donors, and serine-derived glycine and formate are required for backbone synthesis, and mitochondrial metabolic products such as formate, serine-derived glycine, and TCA-cycle intermediates supply further requirements, especially in rapidly proliferating cells. 

Presumably due to the heavy molecular and energetic requirements of purine synthesis, the enzymes of the pathway cluster near mitochondria and microtubules to form purinosomes [15,16,86]. Purinosome assembly and disassembly are highly dependent upon purine availability, with purinosomes forming in response to higher metabolic demands and depleted cellular purine levels [2,15,87]. Collected next to the mitochondria, the purinosome can take advantage of the high concentration of mitochondrially generated ATP and substrates to promote flux through the pathway, as well as minimizing diffusion-related loss of intermediates and throughput time. While the role of the purinosome in cancer has yet to be fully explored, its central position in pathways critical to cell growth makes it an attractive candidate as a potential therapeutic target, and its dependence on mitochondria makes targeting mitochondria a potential means of inhibition of purine biosynthesis. Indeed, disruption of purinosome formation via chaperone inhibition has a synergistic effect with disruption of one-carbon metabolism by the well-established anti-folate drug methotrexate [86]. Conversely, targeting the mitochondrial folate pathway via *MTHFD2* disrupts the production of purines needed for cell growth [62,81]. 

**Figure 3:**
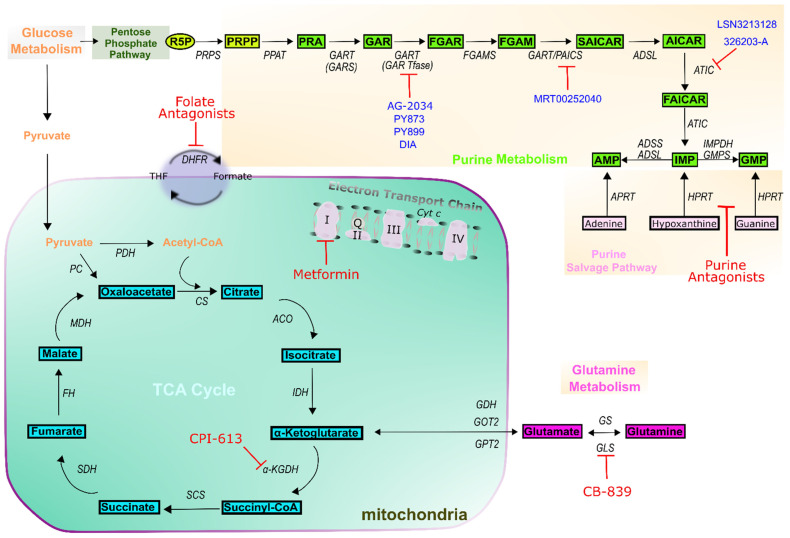
Targeted metabolic inhibitors. The schematic representation shows the metabolic vulnerabilities of cancer. The identification of rational combinations of mitochondrial inhibitors with the standard of care treatment including folate and purine antagonists may bring new insights for cancer treatment. FDA-approved drugs to treat cancer are indicated in red, and drugs under study or in clinical trials are indicated in blue. The identification of novel biomarkers and drug targets to improve the detection and treatment of cancers by modulating purine metabolism is now subject to investigation in several research programs.

The demonstration of the presence of anti-apoptotic proteins in mitochondria suggests another potential target for cancer therapy [88], as does evidence that mitochondrial metabolism is required for tumor growth, indicating that targeting mitochondrial biosynthetic, bioenergetic, and redox functions may be effective in treatment [89]. Many classes of mitochondria metabolic-targeting drugs already exist and are not limited to cancer treatments; these include antimicrobial agents, antidiabetic drugs, and classic anticancer agents. As such, drugs targeting the mitochondria for other purposes may also act to suppress cancer growth, and metformin (electron transport chain inhibitor), CPI-613 (TCA-cycle inhibitor), and CB-839 (glutaminolysis inhibitor) are currently in clinical trials. Both CPI-613 and CB-839 act by inhibiting anaplerotic restoration of TCA cycle intermediates (Figure 3). With glutamine and aspartate generated from intermediates of mitochondrial TCA-cycle metabolism and used as co-substrates of purine biosynthesis enzymes, inhibiting mitochondrial metabolism may potentially be effective to decrease the purine biosynthesis levels in proliferating cancer cells [32]. Similarly, the biguanide metformin is a putative mitochondrial ETC complex I inhibitor and has been shown to suppress nucleotide levels in cancer cells [29]. However, there is a need for more comprehensive studies to determine the direct effect of mitochondrial drugs on purine biosynthesis in cancers and how to balance inhibition of cancer growth with the dependence of all cells, not just cancer cells, on mitochondrial output [89].

## 6. Mitochondria and Purine Metabolism in Ovarian Cancer

The microenvironment-dependent metabolism in OvCa plays an important role in the tumor biology and progression of the diseases [90]. Increased fatty acid metabolism and glycogen accumulation due to hypoxic conditions are metabolic hallmarks of chemoresistance in OvCa [91]. Notably, an important feature of OvCa phenotype is the marked dependence of glutamine metabolism rather than glucose [73]. Mounting evidence shows that highly invasive OvCa lines are glutamine-dependent, and high expression of genes involved in glutaminolysis and mitochondrial TCA-cycle metabolism correlates with poor patient survival rates. More recent work suggests that, as with other cancers, de novo glutamine metabolism serves as a nitrogen donor for biosynthetic purposes through the activity of *GS* and is necessary to drive OvCa growth: specifically, targeting stromal *GS* activity and *GLS* activity in ovarian cancer cells reduced tumor growth and metastasis [92]. In the case of particularly chemoresistant ovarian cancers, this approach can be combined with others, such as inhibition of PARP-dependent genome repair [93].

Metabolic reprogramming toward the mitochondrial Oxidative Phosphorylation (mt-OxPhos) pathway rather than glucose metabolism suggests a therapeutic regimen of treating OvCa with antidiabetic drugs such as metformin. Metabolomic analysis of the mechanism of metformin in ovarian cancer using patient samples confirmed that the predominant anti-tumorigenic effect of metformin is driven by targeting tumor-cell-intrinsic mitochondrial metabolism [30,32]. Moreover, a stage II clinical trial in ovarian cancer demonstrated better overall survival in the metformin-treated group compared with untreated [31]. The close connection between mitochondrial glutamine metabolism and purine biosynthesis therefore suggests that manipulation of the latter may provide an additional means of treatment of these difficult cancers. Recent work has demonstrated that OvCa features altered nucleotide metabolism, treatable with a combination of upstream inhibition of the MAP kinase master regulator, and downstream inhibition of altered chromatin remodeling and resulting in reduced levels of nucleotide synthesis enzymes and intermediates [94]. However, more comprehensive studies remain necessary to characterize mechanisms connecting upregulation of purine biosynthetic proteins to ovarian cancer prognosis.

## 7. Outlook on Novel Purine-Based Cancer Treatment Methodologies

We have discussed how antimetabolites and drugs otherwise targeting mitochondrial metabolism act on different types of cancer as primary treatments. Enzymes involved in the purine de novo biosynthetic pathway are among the most frequently overexpressed proteins across cancers. Accordingly, purine antimetabolites are widely used as an effective cancer therapy, acting to inhibit purine metabolism by inactivating purine biosynthesis enzymes, thereby disrupting nucleic acid synthesis and stalling the energy supply to the cell [95]. However, the heterogeneity of individual cancers, tendency to acquire chemoresistance under selective pressure, and frequent toxic side effects highlight the need for continued research and drug development. As an example to illustrate the latter point, the *GART* inhibitor lomotrexol showed marked side effects in clinical trials and failed to gain FDA approval for use in cancer treatment [96]. However, extensive work continues with improving outlook. While side effects remain common, another *GART* inhibitor, AG2034, showed improved inhibition of tumor growth [97], and more recently developed inhibitors, PY873, PY899, and DIA have been under investigation [28,98,99]. To investigate other enzymes in the pathway, a virtual ligand screen of the National Cancer Institute Diversity compound set was used to identify a novel ATIC inhibitor, 326203-A. This compound was demonstrated to be capable of binding a subunit AICAR transformylase (*AICARFT*) of *ATIC* [100]. Similarly, significant growth inhibition by another novel AICARFT inhibitor, LSN3213128 has been observed in xenograft breast and lung cancer models [101]. The overexpression of *PAICS* is common to many forms of cancer and recognized as a potential target for inhibition [34,102,103]. The small molecule MRT00252040 has shown potential early promise as an inhibitor [104] (Figure 3).

*TGART*, *ATIC*, and *PAICS*, of course, are only a fraction of the targetable purine biosynthetic pathway. Clinical and experimental work has shown that that the overexpression of other pathway enzymes, notably *PPAT* and *FGAMS*, is associated with poor liver cancer survival rates [105]. Furthermore, the colocalization of the purinosome with mitochondria suggests an additional set of important interactions that may serve as targets for disruption, with the potential for a more tightly targeted effect on cancer cells with hyperactive mitochondria relative to untransformed cells [16,106]. However, the characterization of purinosome formation and localization is still in its early stages, and important questions remain. The mechanisms of signaling and regulating the formation of the purinosome are unclear, as are the means by which purinosomes localize to the mitochondria. Furthermore, effective treatment requires detailed knowledge of how this process varies between different forms of cancer. There is still a need for the development of novel cancer treatments with reduced side effects and different mechanisms of action, and the intersection of mitochondrial respiration with purine biosynthesis is an attractive candidate for each.

## Figures and Tables

**Table 1:** Antimetabolites and mitochondria metabolic-targeting drugs to treat malignancies.

Drug Class	Drug Name	Target(s)	Example Indications	Reference
Folate antagonists	Aminopterin	Dihydrofolate reductase (*DHFR*)	Leukemias	[26,27]
	Methotrexate	*DHFR* Thymidylate synthase (*TS*) Bifunctional purine biosynthesis protein PURH (*ATIC*) Amido phosphoribosyltransferase (*PPAT*)	Acute lymphoblastic Leukemias (ALL) Lymphoma Brain tumors Osteosarcoma Breast cancer	[26,27]
	Pemetrexed	*DHFR* *TS* *ATIC* Trifunctional purine biosynthetic protein adenosine-3 (*GART*)	Lung cancer Ovarian cancer Head and neck Liver cancer Mesothelioma Advanced cancers	[26,27,28]
	Pralatexate	*DHFR* *TS*	Multiples Myeloma (MM)	[26,27]
Purine antagonists	6-mercaptopurine	Hypoxanthine-guanine phosphoribosyltransferase (*HGPRTase*) *PPAT*	Leukemias ALL	[23,24]
	6-thioguanine	*HGPRTase*	Leukemias ALL	[23,24]
	Fludarabine Cladribine	DNA synthesis DNA repair	Leukemias MM ALL	[23,24]
	Pentostatin	Adenosine deaminase (*ADA*) DNA synthesis	Leukemias ALL Renal cancer	[23,24]
	Clofarabine Nelarabine	DNA elongation	Leukemias ALL Recurrent neoplasm	[23,24]
Mitochondria	Metformin	ETC complex I	Ovarian cancer Breast cancer Prostate cancer Endometrial Lung cancer	[29,30,31]
	CPI-613	α-ketoglutarate Dehydrogenase (*α-KGDH*)	Pancreatic cancer AML	[32]
	CB-839	Glutaminase (*GLS*)	Lung cancer	[32]

## Data Availability

Not applicable.
